# Loss of Notch1 Disrupts the Barrier Repair in the Corneal Epithelium

**DOI:** 10.1371/journal.pone.0069113

**Published:** 2013-07-18

**Authors:** Asadolah Movahedan, Neda Afsharkhamseh, Hossein M. Sagha, Jarna R. Shah, Behrad Y. Milani, Farnoud Y. Milani, Hercules D. Logothetis, Chi-Chao Chan, Ali R. Djalilian

**Affiliations:** 1 Department of Ophthalmology and Visual Sciences, University of Illinois at Chicago, Chicago, Illinois, United States of America; 2 National Eye Institute, National Institutes of Health, Bethesda, Maryland, United States of America; UCL Institute of Child Health, United Kingdom

## Abstract

The corneal epithelium is the outermost layer of the cornea that directly faces the outside environment, hence it plays a critical barrier function. Previously, conditional loss of Notch1 on the ocular surface was found to cause inflammation and keratinization of the corneal epithelium. This was in part attributed to impaired vitamin A metabolism, loss of the meibomian glands and recurrent eyelid trauma. We hypothesized that Notch1 plays an essential role in the corneal epithelial barrier function and is a contributing factor in the pathologic changes in these mice. Notch1 was conditionally deleted in adult Notch1^flox/flox^, K14-Cre-ERT^+/-^ mice using hydroxy-tamoxifen. The results indicated that conditional deletion of Notch1 on the ocular surface leads to progressive impairment of the epithelial barrier function before the onset of corneal opacification and keratinization. Loss of the barrier was demonstrated both by an increase in *in vivo* corneal fluorescein staining and by enhanced penetration of a small molecule through the epithelium. Corneal epithelial wounding resulted in significant delay in recovery of the barrier function in conditional Notch1^-/-^ mice compared to wild type. Mice with conditional deletion of Notch1 did not demonstrate any evidence of dry eyes based on aqueous tear production and had normal conjunctival goblet cells. In a calcium switch experiment *in vitro*, Notch1^-/-^ cells demonstrated delayed membrane localization of the tight junction protein ZO-1 consistent with a defect in the epithelial tight junction formation. These findings highlight the role of Notch1 in epithelial differentiation and suggest that intrinsic defects in the corneal epithelial barrier recovery after wounding is an important contributing factor to the development of inflammatory keratinization in Notch1^-/-^ mice.

## Introduction

The corneal epithelium is the outermost layer of the cornea that is in direct contact with the outside environment. It is a 4 to 5-cell-layer stratified non-keratinized squamous epithelium that is essential for corneal clarity and hence vision. Functionally, it is an integral component of the ocular defense system where it provides a protective barrier for the cornea against pathogen invasion. Likewise, it plays an important regulatory function in the passage of solutes and macromolecules [1]. The corneal epithelial barrier function, like many other epithelial tissues, is determined by its differentiation program which in part regulates the epithelial cell-cell junctions, most importantly the tight junctions. Tight junctions have been demonstrated in the most differentiated superficial layers of the corneal epithelium and loss of the tight junctions strongly correlates with the loss of the barrier [2-4]. In addition to the tight junction structures, the corneal epithelial barrier is also dependent on the integrity of the ocular surface tear film as well as the expression of mucins on the surface epithelium.

The corneal epithelial barrier function is compromised in nearly all ocular surface disorders ranging from dry eyes to severe corneal infections and ulcerations [5,6]. An ineffective corneal epithelial barrier not only increases the risk of infections but also can lead to break down of the epithelium which can results in scarring or melting of the corneal stroma with significant loss of vision. Currently, there are very few specific treatments to enhance the corneal epithelial barrier function.

Many of the regulatory mechanisms governing corneal epithelial barrier function have been studied before [7,8]; however the role of Notch signaling in this process has not been completely defined. The Notch signaling pathway, a well-known cell-fate determination pathway during development, has also been implicated in a number of important cellular functions in adult tissues including differentiation, proliferation and migration [9-12]. This cell to cell signaling mechanism involves membrane bound Notch receptors (Notch 1-4) and corresponding membrane bound ligands, Delta (Delta 1, 2 and 4) and Jagged (Jagged1 and 2). Upon ligand binding, the Notch receptor is externally cleaved by ADAM (a disintegrin and metalloproteinase) and subsequently internally cleaved by the γ-secretase complex. This sequence releases the Notch intracellular (IC) fragment that travels to the nucleus and associates with CBF1/RBPJκ trans-activating target genes including Hairy/Enhancer of Split (Hes) (canonical pathway). Downstream effectors such as Deltex mediate the effects of Notch in the non-canonical pathway [13].

The role of Notch in corneal epithelial development, differentiation, and proliferation has been examined [14-25]. Recently Zhang et al used a reporter mice to map the cells where Notch1 had been activated during the development of the ocular surface [24]; they found that cells with activated Notch1 were present in the eyelid, conjunctiva and corneal epithelium at embryonic day 15 and postnatal day 1, however by day 30 it was preferentially restricted to the conjunctiva. Using immunohistochemistry for Notch1IC, we reported that by postnatal day 30, when the epithelium is mature, there is expression of Notch1IC throughout the cornea in the basal and immediate suprabasal layers [16]. Among the various Notch receptors, Notch1 is the most well studied subtype in the cornea as mice with conditional loss of Notch2 do not have any corneal phenotype [26]. The critical role of Notch1 in the corneal epithelium was first highlighted in a report by Nicolas et al, who showed that deletion of Notch1 under the keratin 14 promoter leads to progressive inflammation and keratinization of the central cornea [15]. Later, Vauclair et al. reported vitamin A metabolism and recurrent epithelial trauma due to meibomian gland loss as the underlying mechanism for the development of keratinization [14]. Additional studies have also implicated Notch1 in clinical manifestation of ocular surface disease; in particular, a reduction of Notch1 expression was demonstrated in the conjunctival cells of patients with dry eyes [25].

Recently, we reported a role for Notch signaling in corneal epithelial cell migration during corneal wound healing. Specifically, we demonstrated that Notch1 is reduced in the leading edge of corneal epithelium during wound healing which in turn enhances the migratory behavior of corneal epithelial cells [22]. In the current study, we developed conditional Notch1 knockout mice and carefully evaluated the involvement of factors such as meibomian glands, goblets cells and lacrimal gland in the phenotype development. We identified a previously unrecognized role for Notch1 in corneal epithelial barrier recovery after wounding, providing further insight into the underlying pathophysiologic mechanisms of ocular surface diseases with barrier impairment.

## Methods

### Development of Conditional *Notch1* ^*-/-*^ mice

All the animal experiments were conducted in compliance with the recommendations of the Association for Research in Vision and Ophthalmology (ARVO). The protocol was approved by the Committee on the Ethics of Animal Experiments of the University of Illinois at Chicago (Protocol Number: 11-183). All surgeries were performed under general anesthesia, and all efforts were made to minimize suffering.

We developed mice with conditional deletion of Notch1 in the surface epithelium similar to that described earlier by another group [14,15]. We used Notch1 flox/flox mice (B6.129X1-Notch1^tm2Rko^/GridJ, The Jackson Laboratory, Bar Harbor, Maine, USA) in which *loxP* sites flank exon 1 of the Notch1 gene [27]. To conditionally delete Notch1, we used K14-Cre-ERT mice expressing Cre-ERT under the keratin14 (K14) promoter (KRT14-Cre/ERT) 20Efu/J, The Jackson Laboratory) as previously described [28]. Cre-ERT is a Cre-recombinase that has been fused with an estrogen receptor which upon binding of tamoxifen, is translocated into the nucleus. In these mice, the expression of Cre-ERT is targeted to epithelial tissues that express K14, a marker of basal (undifferentiated) epithelial cells. On the ocular surface, K14 is expressed in the basal layer of the corneal and conjunctival epithelium as well as the epithelial linings of both lacrimal and meibomian glands.

We first mated Notch1^flox/flox^ with K14-Cre-ERT^+/+^ mice to obtain the double heterozygote Notch1 Notch1^flox/+^, K14-Cre-ERT^+/-^ mice. These mice were then back-crossed with Notch1 Notch1^flox/flox^ mice which, as expected by Mendelian ratio, resulted in ¼ having a genotype of Notch1^flox/flox^, K14-Cre-ERT^+/-^. Notch1 was conditionally deleted in 2-4 month old Notch1^flox/flox^, K14-Cre-ERT^+/-^ mice with 3-5 consecutive days of either intraperitoneal injection (1 mg/20 g body weight) or topical (0.1 mg/ml dissolved in mineral oil) application of 4-hydroxytamoxifen (4-OHT).

### Immunostaining

Immunostaining of mouse eye cryo-sections or cultured mouse corneal epithelial cells were performed according to our previously published protocol [29] using the following antibodies: polyclonal rabbit anti-K10 (Covance, Princeton, NJ, dilution 1:500), rat anti-zonula occludens (ZO)-1 (R-26-4C, Dep. of Anatomy and cell biology, Harvard Medical School, Boston, MA – obtained through Developmental Studies Hybridoma Bank, University of Iowa – dilution 1:10), FITC conjugated anti-rabbit and anti-rat IgG (dilution 1:250; both from Jackson Immunoresearch, West Grove, PA). The sections were examined using a spinning disc confocal microscope (Z1, Carl Zeiss, Thornwood, NY), and photographed with an AxioCam (Carl Zeiss) camera.

### Western Blots

Western blots were performed as previously described [22]. The following antibodies were used: rabbit anti-cleaved-Notch1 Val1744 (Cell Signaling, Danvers, MA, dilution1:500), monoclonal rabbit anti-GAPDH (Cell Signaling1:5000) and monoclonal rabbit anti-Notch1 (D1E11) xp (Cell Signaling, dilution 1:500). Detection was performed by ImageQuant LAS 1040 detection system and quantified using ImageQuant software (both from GE Healthcare, Piscataway, NJ).

### Histology

Hematoxylin and eosin (H&E) staining was performed according to previously published methods [30]. Oil Red O staining was used to visualize the lipids (in the meibomian glands). This was done using cryo-sections fixed in neutral buffered 10% formaldehyde (Sigma-Aldrich, St. Louis, MO) for 20 minutes. Slides were then placed in 60% 2-propanol and incubated in pre-warmed 0.5% Oil-red-O stain for 15 min, rinsed again in 60% 2-propanol, counterstained with 10 dips in Meyer’s hematoxylin and rinsed in distilled water and then mounted [31].

### *In vivo* Biomicroscopy and Fluorescein Staining

Slit lamp biomicroscopy and photography was done using a Nikon FS-2 photo-slit lamp with a Nikon D200 camera (Melville, NY). Corneas were stained with 10 µL of 1% fluorescein sodium (Akron, Lake Forest, IL) diluted in phosphate buffered saline (PBS) and photographed using the same system under blue filter.

### EZ-Link Sulfo-NHS-LC-Biotin Barrier Function Test

The barrier function was assessed following a previously published protocol [32]. Briefly, prior to euthanasia, 30 µL of a 10 mM solution of EZ-Link-Sulfo-NHS-LC-Biotin (Thermo Scientific, Rockford, IL) was applied to the mouse eyes. After 15 minutes, the eyes were extensively rinsed with PBS before enucleation. Cryo-sections prepared, were fixed in acetone and incubated in 10–20 µg/ml solution of rhodamine conjugated streptavidin (Jackson Immunoresearch) for 15 minutes and then washed extensively with PBS before counterstaining with DAPI. The sections were examined using a spinning disc confocal microscope (Z1; Carl Zeiss, Jena, Germany), and photographed with an AxioCam camera (Carl Zeiss).

### Mouse Corneal Epithelial Cell Culture

Corneal epithelial cells were isolated from mouse eyes following previously published methods [33]. In brief, after induction of euthanasia, the whole eyes were removed and washed profusely first in PBS and gentamicin reagent solution 50 mg/ml (Life Technologies, Gibco, Grand Island, NY, 1:5000 in PBS) for 5 minutes followed by 5 minutes of rinsing with PBS for four times. The globes were then submerged in 10 mg/ml Dispase (Life technologies) solution in DMEM/F12 (1:1) with 36 mg/ml sorbitol (Fisher Scientific, Pittsburgh, PA) and kept in 4°C for 16 hours. To separate the epithelial sheets, the corneal epithelium was peeled off using a small scraper, then the epithelial sheets were digested in 0.25% trypsin without EDTA (Life Technologies) at 37°C for 10 minutes and neutralized with soybean trypsin inhibitor 2 mg/ml solution (Life Technologies). The sheets were pipetted multiple times before centrifugation in 4°C at 800 g for 5 minutes. The supernatant was removed and the cells were re-suspended in defined keratinocyte serum-free medium (D-KSFM, Life Technologies) and plated in collagen coated tissue culture plates or chamber slides. Cells were treated with 1 µM 4-OHT, diluted in D-KSFM for 48 h to induce Notch1 deletion.

### Aqueous Tear Production Measurement

The mice were placed under anesthesia by ketamine (100 mg/kg) and xylazine (5 mg/kg). Immediately following, 2 cm pieces of phenol red threads (Zone-Quick, Menicon, San Mateo, CA) were positioned in the lateral canthus. The wet (red colored) segment of the thread was measured in millimeter after 30 seconds [34].

### Conjunctival Impression Cytology

After euthanasia, eyelids of four WT and four Notch1^-/-^ mice were excised, flattened and placed epithelial side down on a dry glass slide, pressed against it with gentle pressure for 5 seconds and then peeled off slowly after 2 minutes. The remaining cells on the slide were fixed in 10% formaldehyde and stained with PAS as described earlier. For quantifications the number of cells were counted in 10 random microscopic fields with 20X magnification for each sample. The ratio of goblet cells to epithelial cells was compared between the two groups.

### Mouse Corneal Epithelial Wounding

A 2.0 mm central corneal epithelial wound was made by scraping the corneal epithelium in both WT and Notch1^-/-^ mice as described before [26]. Corneal fluorescein staining and barrier function was examined using slit lamp microscopy and EZ-Link-Sulfo-NHS-LC-biotin test at 24 hour intervals.

### Statistical Analysis

Statistical Package for Social Sciences (SPSS) software V 13.0 (SPSS Inc., Chicago, IL) was used for data analysis. For analysis of fluorescein staining and LC-biotin penetration Fisher’s exact test was used. Chi-square test was used to analyze the difference between Notch1^-/-^, Notch1^+/-^ and WT in the development of corneal opacity and keratinization. To compare the difference in the percentage of goblet cells, mean fluorescein staining, aqueous tear production and intensity of Zo-1 staining Student’s t-test was used. Experiments were replicated at least three times and for each immunohistologic experiment a minimum of 3 sections were analyzed. Animal experiments were performed on age-matched groups within an experiment.

## Results

### Notch1 deletion in the corneal epithelium leads to progressive barrier impairment

**Figure 1:**
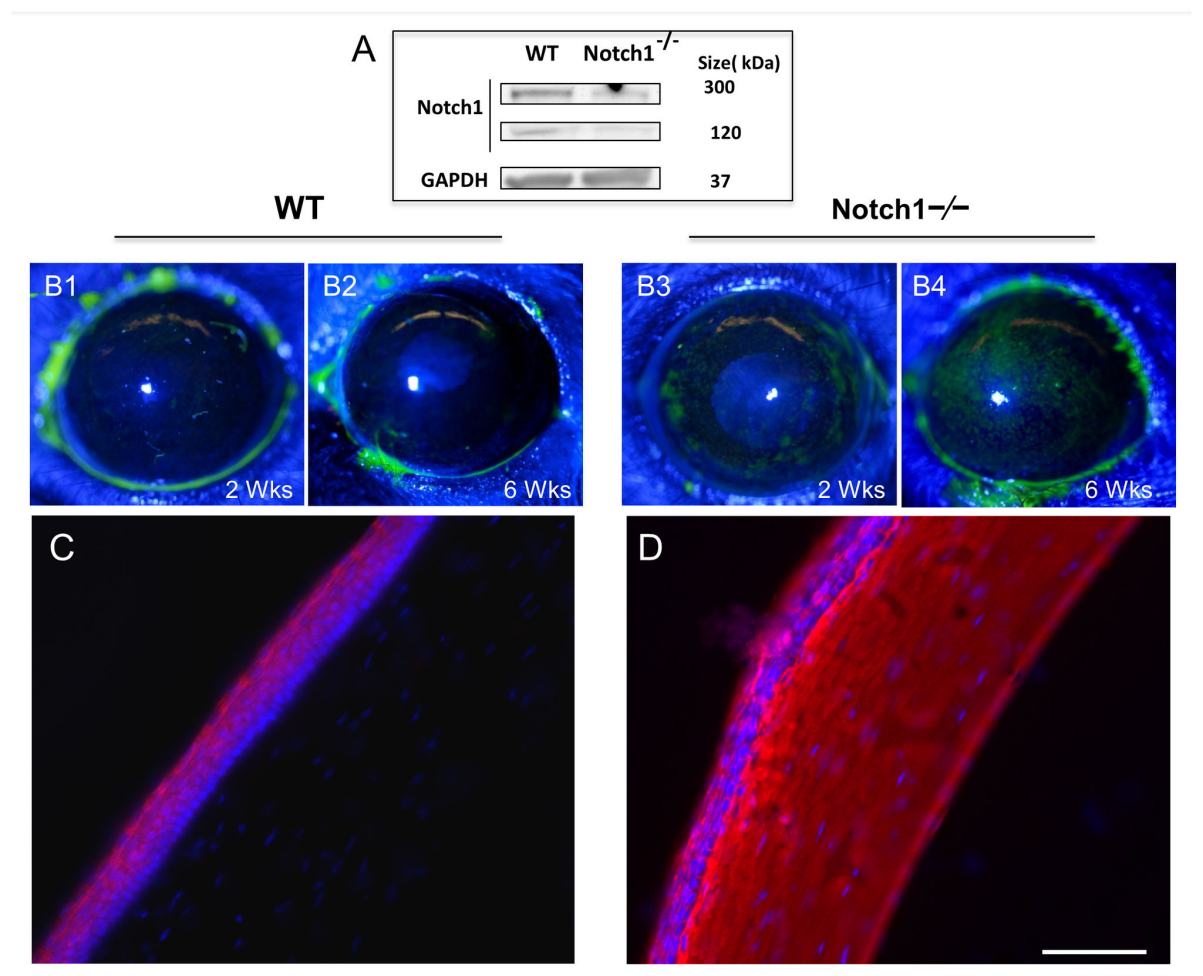
Notch1 deletion in the corneal epithelium leads to progressive barrier impairment. Notch1 knockout efficiency was evaluated by western blotting of pooled corneal epithelial sheets (N=8 per group) from conditional Notch1^-/-^ mice showing 64% to 70% decrease in Notch 1 expression comparing to epithelial sheets from WT littermates (A). Compared to WT (B1, B2), Notch1 deleted corneas demonstrate increased fluorescein staining at 2 (B3) and 6 (B4) weeks after 4-OHT treatment. LC-biotin (stained red with rhodamine) could not penetrate beyond the top few layers of the epithelium in WT mice (C) while it passed the epithelium and reached the stroma in Notch1^-/-^ mice at 4 weeks after Notch1 deletion (D). Red: rhodamine. Blue: DAPI; Scale bar: 50 µm.

**Figure 2:**
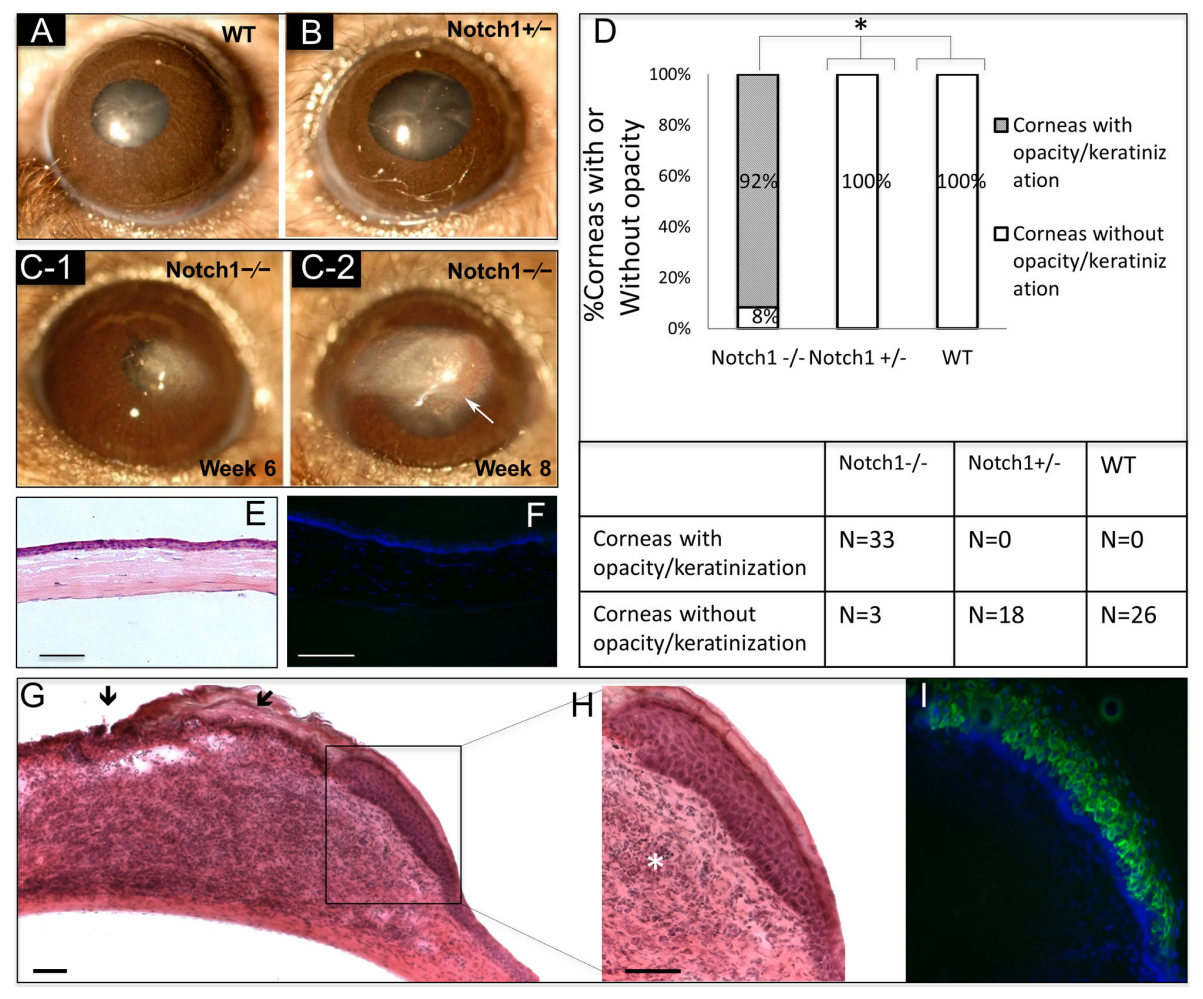
Notch1^-/-^mice develop progressive corneal opacification and keratinization. In WT (A) and Notch1 heterozygotes (N1^+/-^) mice (B) corneal examination does not show any change after 4-OHT treatment and stays normal while conditional Notch1^-/-^ mice at 6 weeks after tamoxifen injection, demonstrate early corneal stromal opacification (C-1) which later progressed to keratinization and secondary neovascularization (white arrow head) (C-2). By 8 weeks after treatment almost 92% of Notch1^-/-^ mice developed corneal opacification/keratinization, while none of the WT or Notch1^+/-^ group showed this phenotype (*P* < 0.0001) (D). Wild type mouse corneal section showing normal histology on H&E staining (E) with no evidence of keratin 10 by immunostaining (F). On H&E staining, corneal lesions in Notch1^-/-^ mice demonstrate significant stromal infiltration (asterisk) with areas of keratinization and ulceration (black arrows) (G, H). The expression of keratin 10 (green) in the metaplastic epithelium confirms the skin-like epithelial phenotype (I). Green: FITC, Blue: DAPI; Scale bar: 50 µm.

Notch1 was conditionally deleted in 2-4 months old Notch1^flox/flox^, K14-Cre-ERT^+/-^ mice following intra-peritoneal injection of 4-OHT for 3-5 consecutive days. Efficiency of Notch1 deletion was estimated by western blot on a pooled sample of corneal epithelial sheets (N = 8 per group) and found to be 64-70% (Figure 1A). No loss of Notch1 or any phenotype was ever observed in untreated mice confirming that Cre-ERT was not spontaneously activated. Likewise, no phenotype was ever noted in Notch1^+/-^ heterozygotes (Figure 2B).

The mice were examined weekly after completing the treatment with 4-OHT. Typically by 2 weeks after Notch1 deletion, the mice continued to demonstrate clear corneas by slit lamp examination. However, fluorescein staining, which measures the integrity of the corneal epithelial barrier, began to show increased staining (uptake) compared to control littermates -- which as a control measure had also been treated with 4-OHT (Figure 1 B1–B4). The corneal staining progressively increased every week. The degree of staining after Notch1 deletion was further quantified in the central 1.5 mm of the cornea on a scale of 0 to 3 [35]. At 2 weeks after tamoxifen injection 62.5% (5/8) eyes and at 6 weeks 100% (8/8) had developed variable degrees of scattered corneal fluorescein staining which was highly significant compared to WT eyes with no staining in all subjects (N = 8) after 2 or 6 weeks (*P*= 0.026). The mean fluorescein staining score at 6 weeks was and found to be 2.5 ± 0.6 in Notch1^-/-^ vs 0.8 ± 0.5 in WT (*P* < 0.01). It should be noted that changes in the barrier were first evident while the corneal stroma remained clear, however by 6 weeks, the stroma also began to develop central opacification.

We proceeded to perform a functional assay of the barrier using NHS-sulfo-LC-biotin test [32]. The results confirmed the fluorescein staining findings by demonstrating increased permeability to LC-biotin and its penetration into the stroma in the conditional Notch1 deleted mice (Figure 1D). In contrast, LC-biotin was unable to penetrate beyond the corneal epithelium of WT mice (Figure 1C).

### *Notch1* ^*-/-*^ mice develop progressive corneal opacification and keratinization

As reported previously [14], approximately 4-6 weeks after conditional deletion of Notch1^-/-^, the mice began to develop opacities in the corneal stroma (Figure 2C-1). By 8 to 10 weeks, 92% (33/36) of corneas had typically progressed to develop skin-like keratinization in the epithelium marked by the expression of epidermal type keratin 10 (Figure 2 I) compared to WT (0/26) or Notch ^+/-^ (0/18) which never developed any keratinization (Figure 2E, 2F) with tamoxifen injection (*P*<0.0001) (Figure 2C-2, 2D). Histologic examination of the cornea demonstrated significant inflammation and areas of ulceration (Figure 2G, 2H). Neovascularization was also frequently observed (Figure 2C-2).

### *Notch1* ^*-/-*^ corneal epithelium is unable to recover its barrier function after wounding

**Figure 3:**
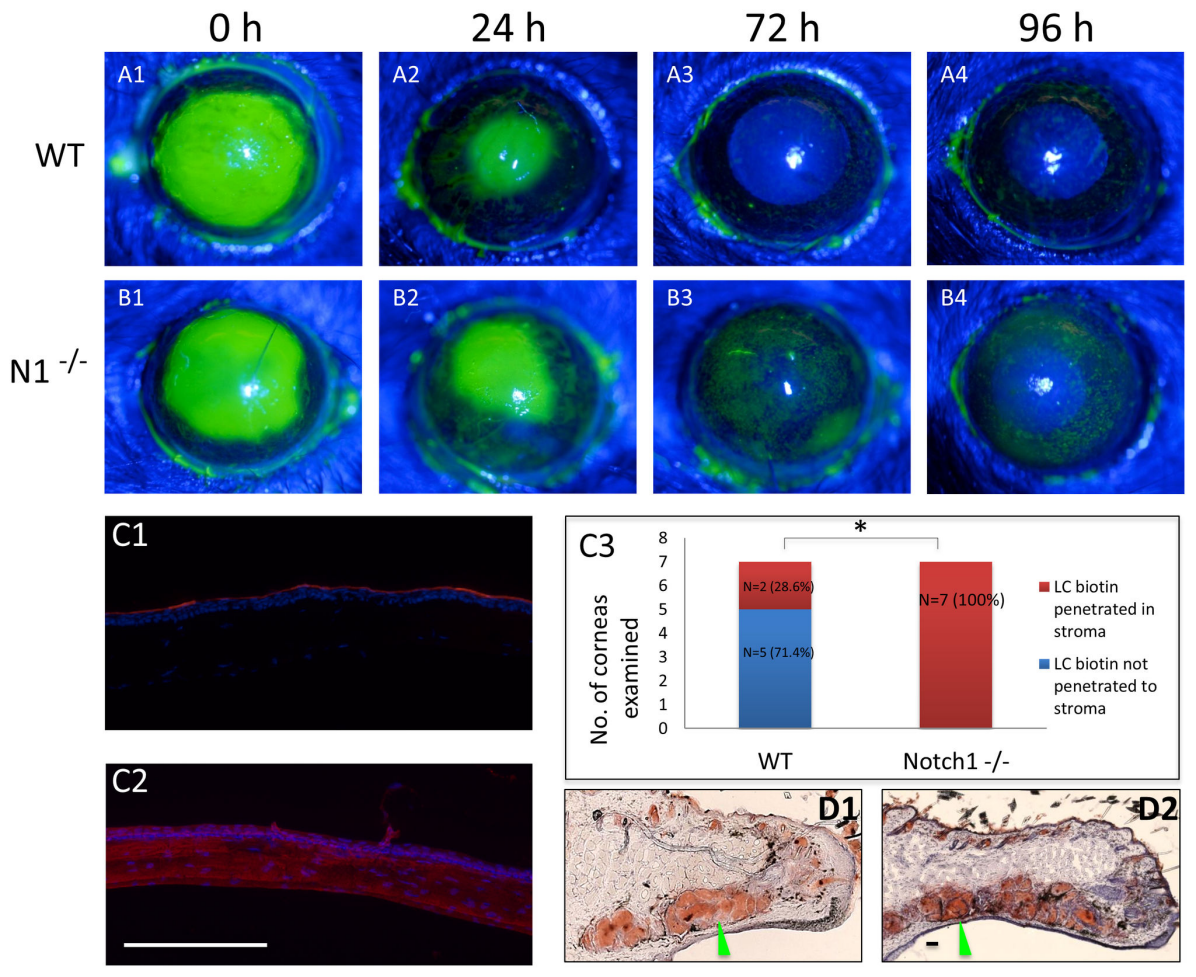
Notch1^-/-^ corneal epithelium is delayed in recovering its barrier function after wounding. Fluorescein staining of WT (A1-A4) and Notch1^-/-^ (B1-B4) eyes immediately (0 h), 24, 72 and 96 hours after 2.0 mm central corneal epithelial debridement wounds; indicating a delay in barrier recovery in Notch1^-/-^ eyes compared to WT. LC-biotin barrier function test at 96 hours post-wounding showing that the WT cornea is impermeable to LC-biotin (C1) while in the Notch1^-/-^ cornea LC-biotin has penetrated into the stroma (C2). LC-biotin staining showed penetration of the molecule into the stroma in 100% (7/7) of Notch1^-/-^ mice compared to only 28.6% (2/7) of WT littermates at 96 hours (*p*=0.021). (C3). Oil Red O staining confirmed the presence of oil producing meibomian glands (green arrow head) in both WT (D1) and Notch1^-/-^ (D2) at one week after 4-OHT treatment. Red: rhodamine, Blue: DAPI; scale bar: 100 µm.

An important finding by Vauclair et al., which implicated recurrent corneal trauma in the corneal pathology, was that epithelial debridement wounds to the Notch1^-/-^cornea accelerated the progression of the phenotype [14]. We reasoned that the regenerated corneal epithelium is unable to immediately recover its barrier function and ends with progressive impairment of the barrier after each wounding episode. The barrier function in WT and conditional Notch1^-/-^ mice after epithelial debridement wounds was therefore assessed. The wounding experiments were performed in mice just 5 days after receiving 4-OHT in order to minimize the contribution of the eyelid pathology. The results indicated a significant delay in epithelial barrier recovery after wounding in Notch1 deleted corneas (Figure 3A–C). In particular, conditional Notch1^-/-^ mice demonstrated persistent fluorescein staining at 96 hours (Figure 3 B4) and penetration of LC-biotin into the stroma (Figure 3 C2) compared to the WT where there was no longer any staining by 72 hours (Fig, 3 A3) and most of the eyes were impermeable to LC-biotin (Figure 3 C1). Analysis of the LC-biotin staining showed penetration of the molecule into the stroma in 100% (7/7) of Notch1^-/-^ mice compared to only 28.6% (2/7) WT littermates at 96 hours post wounding (*P* = 0.021) (Figure 3 C3). The barrier in Notch1^-/-^ mice was found to recover by 144 hours after wounding, indicating that it is ultimately able to reform the barrier however with a significant delay (data not shown).

Previously, it was reported that atrophy of the meibomain glands and secondary eyelid margin keratinization is an important contributing factor to the corneal pathology in Notch1^-/-^ mice [14]. Meibomain glands are holocrine glands located in the upper and lower eyelid that are analogous to sebaceous glands. They produce an oily secretion that contributes to the stability of the ocular surface tear film [36]. The importance of eyelid trauma to the corneal disease was shown in an experiment by Vaulclair et al. where suturing the eyelids closed prevented the development of the keratinized corneal plaques [14]. In order to determine whether the delayed barrier recovery is related to the eyelid pathology, we proceeded to examine the meibomian glands in the same wounded mice. Oil red O staining of the eyelid tissue confirmed that the meibomian glands (N= 5) still produced oil at 1 week after 4-OHT treatment (Figure 3 D1–D2). These findings suggest that the delay in barrier recovery is most likely due to an intrinsic defect in the corneal epithelium and is not directly due to the eyelid pathology. It should be noted that examination of the eyelid histology at later time points (2-3 weeks after 4-OHT) did demonstrate cystic changes and loss of the meibomian glands as reported before (data not shown) [14].

### Aqueous tear production is increased in conditional *Notch1* ^*-/-*^ mice

**Figure 4:**
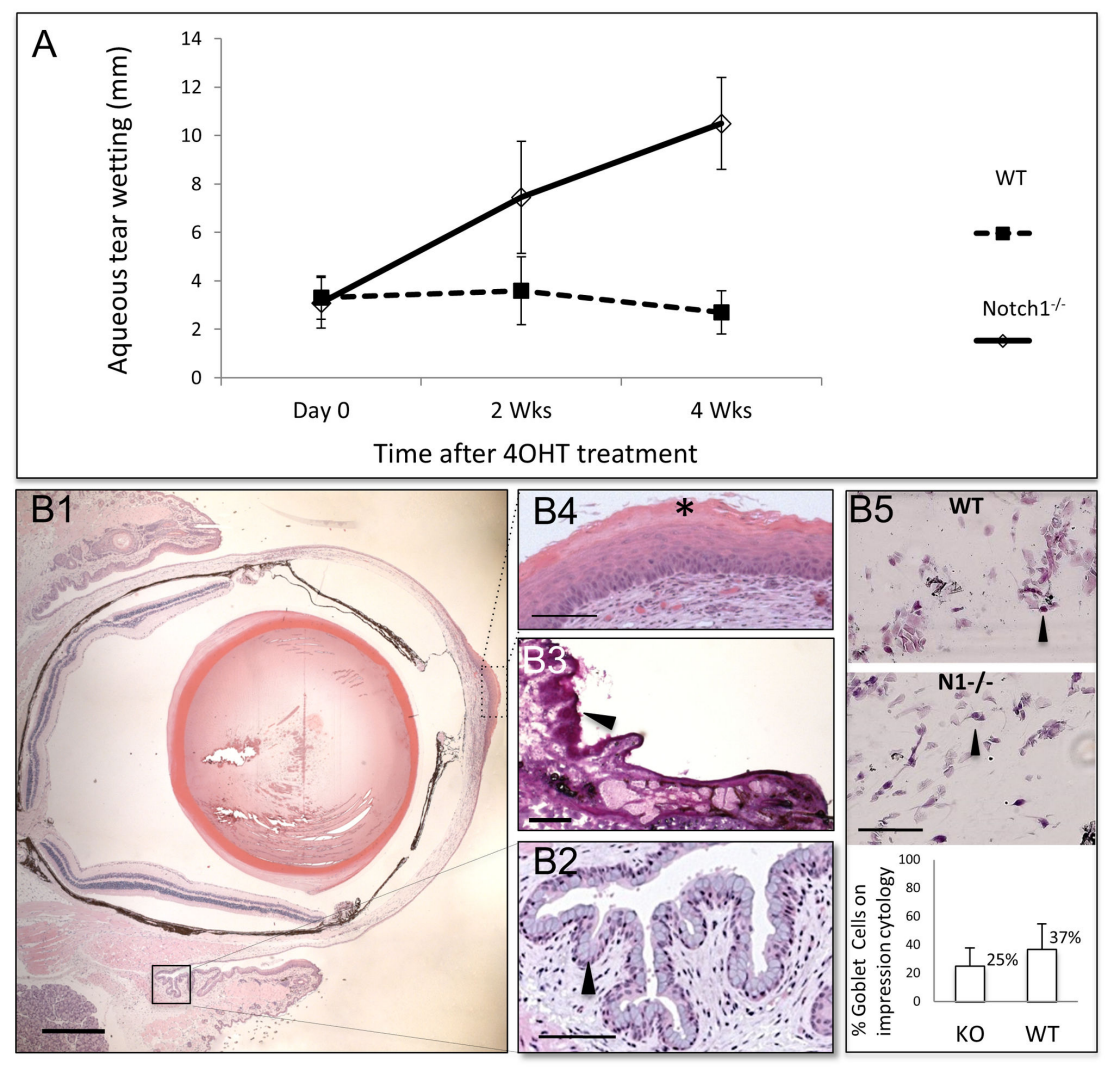
Aqueous tear production is increased and goblet cells are intact in conditional Notch1^-/-^ mice. Aqueous tear measurement by phenol thread test in millimeters at baseline, 2 and 4 weeks after treatment with 4-OHT in Notch1^-/-^ and WT littermates. The mean aqueous tear production in Notch1^-/-^ eyes was found to be significantly higher than WT at 2 (7.4 ± 2.3 mm versus 3.6 ± 1.4, *P* = 0.001) (N=10 per group) and 4 weeks (10.5 ± 1.8 mm for Notch1^-/-^ compared to 2.7 ± 0.9 mm in WTs, *P* <0.001) after the treatment (A). Goblet cells (arrow heads) are visualized by H&E (B1, B2) and PAS (B3) staining in the conjunctival fornix of conditional Notch1^-/-^ mice which have developed keratinization (asterisk) of the central corneal epithelium (B4). Impression cytology of Notch1^-/-^ and WT lids showed no significant difference in the ratio of goblet to epithelial cells (mean ratio of 36.7±18% in N1^-/-^ and 25.1±16.6% in WT, *P* = 0.118). scale bar: 500 µm (B1), 100 µm (B2, B4, B5) and 200 µm (B3).

Given the importance of the tear film in the corneal epithelial barrier function, we proceeded to examine the aqueous tear production in conditional Notch1^-/-^ mice, especially given that keratin 14 is also expressed in the lacrimal gland. We measured the aqueous tear production using phenol red thread test in conditional Notch1 knockout and WT mice both of which had previously been treated with 4-OHT. The mean aqueous tear production at baseline (immediately after 4-OHT treatment) was similar in Notch1^-/-^ (3.09 ± 1.05 mm) and WT (3.31± 0.9 mm) mice (*P* = 0.62). At 2 weeks post treatment, when the cornea was beginning to show barrier impairment, the aqueous tear production in Notch1^-/-^ eyes was found to be significantly higher than WT (7.4 ± 2.3 mm versus 3.6 ± 1.4, *P* = 0.001). At 4 weeks after 4-OHT treatment, the mean aqueous tear production had increased even further to 10.5 ± 1.8 mm for Notch1^-/-^ compared to 2.7 ± 0.9 mm in WTs (*P* <0.001) (Figure 4A). Thus, the aqueous tear production was not only intact, but also seemed to be enhanced in the Notch1^-/-^ mice. Pathologic examination of the lacrimal gland did not reveal any difference between WT and Notch1 knockouts (data not shown).

### Goblet cells are intact in the conditional *Notch1* ^*-/-*^ mice

The production of soluble mucins by the conjunctival goblet cells also contributes to the tear film and plays a central role in maintaining the mucosal phenotype on the ocular surface. Since keratin 14 is also expressed in the conjunctiva, we proceeded to examine the presence of goblet cells in Notch1^-/-^ mice. Histologically, goblet cells were present in the conjunctival fornix in all Notch1^-/-^ eyes including those with advanced corneal pathology (Figure 4 B1–B4). The percentage of goblet cells in the conjunctiva was measured on impression cytology samples and likewise found to be similar (*P* = 0.118) (Figure 4 B5). Therefore, in our model, the loss of Notch1 on the ocular surface did not appear to adversely affect the conjunctival goblet cells.

### Tight junction formation is delayed in *Notch1* ^*-/-*^ corneal epithelial cells *in vitro*

**Figure 5:**
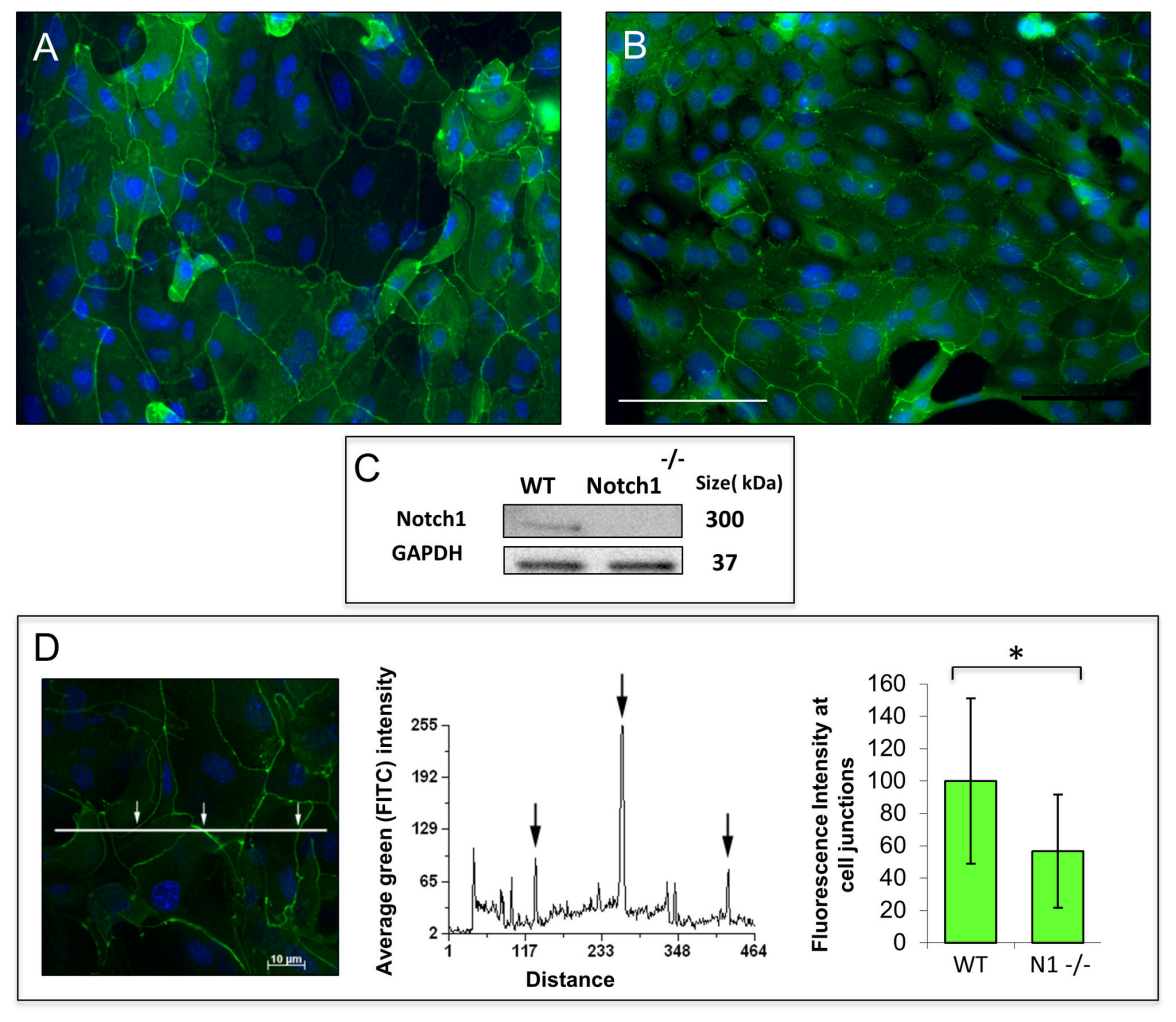
Notch1^-/-^corneal epithelial cells have a delay in tight junction formation in vitro. After 12 hours in 1mM calcium, WT cells frequently showed continuous linear staining at the cell borders indicating more organized tight junctions (A). In contrast, Notch1^-/-^cells exhibited predominantly non-continuous staining at the cell-cell junctions consistent with disorganized tight junction structures (B). Western blot confirmed deletion of Notch1 in knockout cells (C). The mean fluorescence intensity of ZO-1 staining at the cell membranes was measured and found to be higher for WT (arbitrarily set at 100%) compared to Notch1^-/-^ (56%) (D) (N = 119, *P* < 0.001); scale bar: 50 µm.

Given that multiple factors on the ocular surface (e.g. trauma, inflammation) can contribute to the loss of the barrier, we proceeded to examine the expression of tight junctions *in vitro*. Primary mouse corneal epithelial cells from conditional Notch1^-/-^ and WT mice were grown in low calcium serum free media and both treated with 4-OHT for 48 hours. Successful deletion of Notch1 was confirmed by Western blot (Figure 5C). To induce differentiation and tight junction formation, we raised the calcium to 1mM after which the expression pattern of the tight junction protein ZO-1 was examined. At 12 hours after calcium switch, WT cells demonstrated a more continuous staining pattern at the cell-cell junctions (consistent with organized tight junction structures) compared to Notch1 deleted cells which showed a predominance of non-continuous staining pattern (Figure 5A,B). This difference was quantified by measuring the mean fluorescence intensity of ZO-1 staining at the cell membranes using a previously published method [37]. The results indicated a significantly higher intensity of membrane staining in WT (arbitrarily set at 100%) compared to Notch1^-/-^ cells (56%) (*P* <0.001) (Figure 5D). With longer exposure to the high calcium condition (24-48 hours), the Notch1 deleted cells were able to develop a more continuous pattern of ZO-1 staining (mean fluorescent intensity for Notch1^-/-^ was 117.6±32.9 vs 122.4±48.3 for WT, *P* = 0.429) suggesting that tight junction formation can ultimately be compensated by other means *in vitro*.

## Discussion

In this study we have demonstrated an essential role for Notch1 in the corneal epithelial barrier particularly its recovery after wounding. This is consistent with our previous studies which demonstrated that Notch1 is down-regulated during initial stages of wound healing to promote epithelial migration, after which it must be upregulated to induce differentiation and re-establishment of a stratified epithelium and barrier [16,26]. Other studies have similarly highlighted the role of Notch1 in epithelial differentiation [20]. This role for Notch1 in differentiation is similar to that in other stratified epithelia such as the epidermis and intestinal epithelium where loss of Notch1 also leads to an impaired barrier function [38,39]. These results provide additional insight into the phenotype of Notch1^-/-^ mice by highlighting the fact that the barrier impairment may be an important contributing factor in the pathologic changes in the cornea, particularly their development of inflammatory keratinization.

The importance of trauma in exacerbating the barrier is a well described phenomenon in many skin diseases [40]. Trauma appears to “stress” the epithelium and make the barrier impairment become manifest. Immediately after Notch1 deletion on the ocular surface, the corneal barrier does not develop any obvious impairment suggesting that in the absence of trauma (e.g. under closed eyelids), the barrier impairment can be compensated by other means, just as we have shown *in vitro* where the tight junction defect is eventually compensated by other measures. Recurrent epithelial trauma from blinking over time disrupts the epithelial barrier which, in the absence of Notch1, cannot be adequately repaired. We hypothesize that chronic barrier deficiency and trauma in turn activate pathways that contribute to the development of keratinization of the epithelium. As we discuss further below, in this model, both Notch1^-/-^ and Notch1^+^ cells contribute to the keratinized epithelium which suggests that the process of metaplasia is not dependent on the loss of Notch1.

Our *in vitro* results further highlight the intrinsic defect of the corneal epithelium. We focused specifically on tight junctions and found that loss of Notch1 in cultured corneal epithelial cells led to an impairment in tight junction formation. This most likely reflects a more fundamental impairment in the differentiation program and not a specific defect involving the tight junctions. The expression and organization of tight junctions is a highly regulated process that is directed by the differentiation program. Factors such as increased calcium and air-lifting which promote differentiation and stratification also promote tight junction formation. Therefore, we believe that loss of Notch1 is probably not directly affecting tight junctions but rather causing a defect in epithelial differentiation, which also includes formation of tight junctions. A similar defect in tight junction formation was also reported in the 14-3-3σ knockout mice which develop an identical corneal phenotype [41]. Interestingly, the tight junction defect in 14-3-3σ knockout epithelial cells was reversed upon transfection with Notch1IC [41]. Further studies are needed to determine the precise mechanism by which loss of Notch1 leads to impairment of the epithelial differentiation program.

Previously, the phenotype of conditional Notch1^-/-^ mice was partly characterized by Vauclair et al. [14] In particular, they demonstrated the critical role of corneal trauma from eyelids in the development of keratinization. As we have shown in this study, the barrier impairment after trauma precedes the complete loss of meibomian glands and therefore while the eyelid pathology is significant in the progression of the phenotype, it is not required for the barrier defect we observed after wounding. We believe that trauma from normal blinking can stress the epithelium which is further exacerbated by the loss of meibomian glands and eyelid margin keratinization.

A recently published study has reported that loss of Notch function on the ocular surface leads to impaired conjunctival goblet cell differentiation and progressive atrophy of the lacrimal gland [24]. The authors hypothesized that the corneal pathology was secondary to the absence of goblet cell and the aqueous tear deficiency. We did not observe such changes in our mice. As mentioned earlier, we have actually found enhanced aqueous tear production in our conditional Notch1^-/-^ mice. This may be either reflexive tearing due to impaired epithelial barrier or perhaps due to the loss of the meibomian gland function which destabilizes the tear film. The difference between our results and Zhang et al. is most likely due to our use of different mouse models. Specifically, most of their reported findings are with a mouse model that expresses a dominant negative mastermind-like1 (dnMaml1) which inhibits all canonical Notch signaling [42] compared to our study where only Notch1 is knocked out. Although Zhang et al. do report using a conditional Notch1 knockout model for some of their experiments, they used a different driver mouse (*K14-rtTA/TC and tet-O-Cre*) and also deleted Notch1 much earlier by giving doxycycline from P1 to P16, a time when the cornea and ocular surface are still under development [24,43]. In contrast, we deleted Notch1 using K14-Cre-ERT by administering tamoxifen after 2 months of age. Overall, the pathology reported by Zhang et al. [24] maybe somewhat different than that reported by us and Vauclair et al. [14]. In particular, their model shows evidence of dry eye disease with keratinization across the ocular surface (both conjunctiva and cornea) while in our model, keratinization is limited to the central corneal plaques which is more prone to trauma.

Another important point about our mouse model is that it typically leads to a partial and not complete knockout of Notch1. This is mainly because tamoxifen does not activate Cre-ERT in all cells and therefore does not knockout Notch1 in the entire ocular surface epithelium [14,15]. The advantage of using the tamoxifen inducible system is that it allows one to bypass the developmental period and focus on the specific role of Notch1 in adult tissues. However, the fact that it is a partial knockout also highlights an interesting point about the corneal pathology in these mice, namely that complete loss of Notch1 is not required. As shown before by the Kopan group, a complete knockout in the cornea is not necessary for the phenotype and ultimately the keratinized epithelium includes both Notch1^-/-^ and Notch1^+^ epithelial cells [44]. This strongly suggests that the phenotypic switch (i.e. keratinization) may not actually require the loss of Notch1 but rather it may be a consequence of other events on the ocular surface. Therefore, we hypothesize that a threshold level of Notch1 loss in the epithelium leads to impairment of the epithelial barrier function which predisposes it to the pathologic changes due to recurrent trauma. Vauclair et al. identified cellular retinol binding protein 1 (CRBP1) as one of the downstream effectors of Notch1 and hypothesized that impairment in vitamin A metabolism was important for the phenotypic switch. However, the fact that Notch1^+^ cells also contribute to the keratinized epithelium argues against that hypothesis and instead suggests that loss of Notch1 and downstream effectors such as CRBP1 most likely lead to an impairment in the epithelial differentiation and ultimately barrier function.

In summary, we have demonstrated an essential role for Notch1 in the corneal epithelial barrier recovery after wounding. The *in vivo* findings are further corroborated by abnormalities in tight junction formation in Notch1^-/-^ epithelial cells *in vitro*. These results highlight the role of Notch1 in epithelial differentiation and suggest that an intrinsic defect in the corneal epithelial barrier function is an important contributing factor to the development of inflammation and keratinization in these mice. These findings provide further insight into the pathophysiologic mechanisms of ocular surface diseases and suggest Notch signaling may be a potential therapeutic pathway for enhancing the barrier function.
